# Cancer proteomics: from bench to bedside

**DOI:** 10.1038/sj.bjc.6604537

**Published:** 2008-08-12

**Authors:** J F Timms

**Affiliations:** 1 University College London, London, UK

Proteomic technologies hold great promise in the search for clinically useful protein biomarkers for the early detection, diagnosis and prognosis of cancer and for monitoring response to therapy. Moreover, they have the potential for contributing to the discovery of novel drug targets and for testing the biochemical effects of drugs on cells and tissues and to define cancer-related molecular events. This book, from the *Cancer Drug Discovery and Development Series*, provides the reader with a current perspective and anticipated uses of proteomic strategies in cancer therapy as well as basic cancer research.

The book is divided into four parts, beginning with a detailed background on current proteomic technologies. The chapter describes mass spectrometry-based methods and strategies for studying protein phosphorylation and the quantitation of proteins for expression profiling. Although a technical subject, the authors have not overcomplicated the topic, and the chapter provides the non-specialist reader with an understandable background to the methods and also details some of the technical challenges associated with proteomic analysis. The second part of the book plunges the reader into the topic of cell signalling proteomics, with two very specific chapters on the integration of genomics and proteomics for the study of p53 signalling, and the profiling of tyrosine kinases as pharmacological end points for targeted cancer therapy. The general methods and background to the signalling pathways and tyrosine kinase inhibitors with therapeutic use are well described in both chapters, and work in these areas is well-referenced. However, chapter 2 includes an abundance of experimental data from the authors’ own laboratory, drawing the reader away from the focus of the book, and although chapter 3 promises an interesting section on clinical phospho-proteomics, it fails to deliver.

The third part of the book describes clinical applications of proteomics in cancer therapy, although few, if any, proteomic-based tests are actually used in the clinic. This part of the book is perhaps inaccurately entitled ‘Tumour Proteomics’, as it covers an array of topics from oncoproteomics for personalised management, through serum proteomics for cancer detection, to strategies of therapeutic individualisation and target discovery in AML. In chapter 4, the concept of the cancer biomarker is introduced along with the role of proteomics in the discovery of biomarkers for diagnosis, monitoring cancer progression, predicting recurrence, assessing response/efficacy of treatment and selecting patients for given therapies. Several more methodologies and strategies for biomarker discovery are briefly described, although are inadequately referenced. The section is poorly organised and it is not clear how clinically useful such approaches are or will be in the future, particularly for directing personalised therapies. Chapter 5 reads much better, dealing with serum and tissue proteomics and the incorporation of proteomic tests into clinical trials where they are expected to provide useful end points. The introduction describes currently used serum tumour markers, discusses their inadequacies and emphasizes the need for better cancer biomarkers. Several proteomics methods are again described (gel electrophoresis, mass spectrometry, protein arrays, and so on), which would have been better placed in the first section of the book.

Chapters 6–8 provide specific examples of the use of proteomics in the study of renal cell carcinoma, lung cancer and acute leukaemia. Again there are informative backgrounds for the non-specialist reader and several interesting sections on tumour antigens, the importance of sample selection and preparation, molecular diagnosis and classification of cancer using proteomics, multiplexed immune-based protein assays and drug target validation using proteomics. Chapter 9 is excellent and deals with practical aspects of tumour biomarker discovery; a must for all new researchers to the field, although would have been better placed earlier in the book. It describes guidelines for the development and introduction of biomarkers to the clinic and has first-rate sections on pre-analytical, analytical and post-analytical aspects of biomarker assessment and the clinical uses of biomarkers in cancer.

The final part of the book covers bioinformatics and regulatory aspects of proteomics. Chapter 10 provides a useful overview on efforts to annotate the human proteome, references useful data resources and, importantly, highlights the need and global initiatives for standardising the reporting and storage of proteomic information from different sources. The final chapter deals with regulatory issues in the co-development of drugs and proteomic tests. It includes the role of the Food and Drug Administration (FDA) in regulating the use of proteomics in cancer therapy and covers issues of target characterisation, intended use and indication for use, method and operation standardisation and the importance of study design.

Despite the reservations outlined above, the book provides a broad account of the area of cancer proteomics and adequately covers issues relevant to the use of proteomic strategies in clinical research. There are useful reviews of different methodologies, work in different cancer types, on biomarker development and clinical use and on bioinformatics and regulatory aspects of proteomics. The book would be of interest to clinicians and basic cancer researchers alike who are already involved in, or who are considering the use of proteomics in their work. However, the book also reveals that clinical proteomics is still in its infancy, that biomarker research is firmly in the early discovery phase and that there has been little clinical validation of the many recently reported ‘biomarkers’ of cancer. Thus, although proteomics has contributed significantly to our understanding of the molecular biology of cancer, it is a sad fact it has yet to deliver any clinically useful biomarkers.

